# Current Status of Anti-Reflux Surgery as a Treatment for GERD

**DOI:** 10.3390/medicina60030518

**Published:** 2024-03-21

**Authors:** Jooyeon Lee, Inhyeok Lee, Youjin Oh, Jeong Woo Kim, Yeongkeun Kwon, Ahmad Alromi, Mohannad Eledreesi, Alkadam Khalid, Wafa Aljarbou, Sungsoo Park

**Affiliations:** 1 Department of Medicine, Seoul National University Hospital, Seoul 03080, Republic of Korea; 2 Division of Foregut Surgery, Korea University College of Medicine, Seoul 02841, Republic of Korea; inhyeok2@korea.ac.kr (I.L.); kukwon@korea.ac.kr (Y.K.); drmaledrisy@gmail.com (M.E.);; 3 Department of Internal Medicine, John H. Stroger Jr. Hospital of Cook County, Chicago, IL 60612, USA; 4 The Jordanian Ministry of Health, Department of General Surgery, Princes Hamzh Hospital, Amman 11947, Jordan; 5 Taif Armed Forces Hospital, Taif 26792, Saudi Arabia; 6 Dr. Sulaiman Al Habib Hospital, Riyadh 34423, Saudi Arabia

**Keywords:** gastroesophageal reflux, fundoplication, proton pump inhibitors, cost-effectiveness

## Abstract

Anti-reflux surgery (ARS) is an efficient treatment option for gastroesophageal reflux disease (GERD). Despite growing evidence of the efficacy and safety of ARS, medications including proton pump inhibitors (PPIs) remain the most commonly administered treatments for GERD. Meanwhile, ARS can be an effective treatment option for patients who need medications continuously or for those who are refractory to PPI treatment, if proper candidates are selected. However, in practice, ARS is often regarded as a last resort for patients who are unresponsive to PPIs. Accumulating ARS-related studies indicate that surgery is equivalent to or better than medical treatment for controlling typical and atypical GERD symptoms. Furthermore, because of overall reduced medication expenses, ARS may be more cost-effective than PPI. Patients are selected for ARS based on endoscopic findings, esophageal acid exposure time, and PPI responsiveness. Although there is limited evidence, ARS may be expanded to include patients with normal acid exposure, such as those with reflux hypersensitivity. Additionally, other factors such as age, body mass index, and comorbidities are known to affect ARS outcomes; and such factors should be considered. Nissen fundoplication or partial fundoplication including Dor fundoplication and Toupet fundoplication can be chosen, depending on whether the patient prioritizes symptom improvement or minimizing postoperative symptoms such as dysphagia. Furthermore, efforts to reduce and manage postoperative complications and create awareness of the long-term efficacy and safety of the ARS are recommended, as well as adequate training programs for new surgeons.

## 1. Introduction

Gastroesophageal reflux disease (GERD) is a common disease. In 2020, the global prevalence of GERD was 13.98% [1]. In Asia, a meta-analysis conducted in 2020 [2], which included 37 general population-based studies, reported that the prevalence of GERD increased from 11.0% to 15.0% from 2000–2009 to 2010–2019.

“Proven” GERD is defined as a mucosal injury identified through endoscopy and/or abnormal esophageal acid exposure during esophageal pH monitoring. Typical symptoms of GERD include heartburn and regurgitation. In addition, chest pain may present with or without typical symptoms. Hoarseness, chronic cough, and throat clearing can present as extra-esophageal symptoms in patients with GERD [3].

The American College of Gastroenterology (ACG) guidelines for GERD (2021) [3] recommend an 8-week once-daily trial of empirical proton pump inhibitors (PPIs) for patients with typical GERD symptoms without alarming symptoms, including weight loss and gastrointestinal bleeding. However, nearly 40% of the patients treated with PPIs reportedly have persistent symptoms of heartburn and regurgitation [4,5,6]. Refractory GERD is commonly defined as persistent heartburn and/or regurgitation after 8 or 12 weeks of double-dose PPI therapy [7,8]. Furthermore, long-term PPI use has been associated with various adverse effects, such as dementia, osteoporosis, pneumonia, and *Clostridium difficile* infection, in several observational studies [9,10,11,12]. Anti-reflux surgery (ARS) is known to show comparable or superior outcomes compared to medications including PPIs, as identified in a previous meta-analysis of randomized controlled trials (RCTs) [13]. Therefore, the ACG guidelines recommend ARS as a long-term treatment in patients with severe reflux esophagitis (Los Angeles grade C or D), large hiatal hernias, and/or persistent troublesome GERD symptoms with objective evidence of GERD [3].

GERD is usually accompanied by hiatal hernia, displacement of the esophagogastric junction and stomach through the esophageal hiatus. In hiatal hernia, the lower esophageal sphincter is displaced proximally, leading to a mismatch between intrinsic compression of the lower esophageal sphincter (LES) and the extrinsic compression from the diaphragmatic crura, resulting in decreased LES pressure [14]. According to a guideline for hiatal hernia, all symptomatic hiatal hernia should be repaired. Hiatal hernia repair typically involve primary crural closure, mesh reinforcement, and ARS [15].

The Society of American Gastrointestinal and Endoscopic Surgeons guidelines (2021) recommend that ARS may be more beneficial than medical management in patients with chronic or chronic refractory GERD, based on four desirable surgical outcomes: less time with abnormal pH (pH < 4), less post-intervention PPI administration, better short-term quality of life, and better long-term symptom control [16]. The guidelines considered three undesirable surgical outcomes, short-term complications, gas and bloating symptoms, and treatment failure. However, these effects were considered small compared with the desirable effects [16].

Regarding cost-effectiveness, a previous study estimated that ARS was equivalent to medication administration for 8 years after treatment started to become cost-saving at a later stage [17]. Similarly, another cost-effectiveness study, based on the REFLUX trial, reported that surgery was more cost-effective than medication. This was because the anti-reflux effect lasted for at least 5 years after surgery and the reflux symptoms did not worsen postoperatively in patients who did not respond to surgery [18].

Despite these recommendations, ARS is rarely performed. In the United States (US), between 2004 and 2013, ARS was performed in 0.05% of patients with GERD [19]. In England, the rate of ARS was approximately 4.6–5.2 operations per 100,000 people in 2014 [20]. According to National Health Insurance Service data in Korea, ARS was performed on 342 patients from 2012 to 2016, while medication was prescribed to 3.1 million people [21]. Lower ARS rates can be attributed to a lack of interest from both healthcare providers and the general public.

The present narrative review aims to provide comprehensive insights into the efficacy and cost-effectiveness of ARS, as well as its complications, failures, and revision surgeries. It also aims to review the factors that should be considered in determining candidates for ARS and the type of surgery and finally attribute to increase interest in ARS as a treatment of GERD from both physicians and the patients.

## 2. Materials and Methods

We performed a literature review using the PubMed and Google Scholar databases. The search terms used were: GERD, ARS, fundoplication, Nissen fundoplication, Dor fundoplication, Toupet fundoplication, PPI, acid-suppression medication, indication, efficacy, complication, cost-effectiveness, obesity, treatment failure, and revision surgery. Studies published until December 2023 were included in the review, and related articles and bibliographies of the identified articles were also reviewed. All the included articles were screened and reviewed by two authors: J.L. and I.L.

## 3. Efficacy of ARS in Refractory GERD

ARS prevents reflux of gastric material by creating and strengthening the mechanical barrier; thus, in principle, surgery controls refractory GERD symptoms caused by reflux. A previous study has suggested that reflux control through fundoplication is associated with an increase in the mean residual pressure of the LES and a decrease in the frequency of transient LES relaxation [22]. In contrast, other studies have reported that fundoplication is associated with the reinforcement of the gastroesophageal flap valve, which is related to reflux control [23,24].

In patients with “proven” GERD, poor responders to PPI were reported to show significant improvement in GERD symptoms after ARS, which may be attributed to the reduction in acid exposure in the lower esophagus [25]. Surgery significantly decreases the esophageal acid exposure time and the number of reflux events (acidic and weakly acidic), leading to total or subtotal remission of typical GERD symptoms at 3 months after surgery [26]. Additionally, in a well-defined group of patients with functional esophageal disorders, especially in patients with reflux hypersensitivity, ARS showed considerable benefits in symptom remission despite limited evidence [27,28,29].

However, poor responders to PPIs often exhibit poor postoperative symptom control [30,31,32]. Wilkerson et al. [33] reported that symptoms were significantly controlled postoperatively in both good and poor responders. However, the percentage of excellent or good surgical outcomes was lower among poor responders (94% vs. 87%; *p* = 0.08) [34]. One prospective study showed that anatomical improvements after ARS were similar to those observed in PPI responders and non-responders. However, the rates of symptom remission were higher in PPI responders than in non-responders (heartburn: 93% vs. 73%, *p* = 0.01; regurgitation: 96% vs. 84%, *p* = 0.04; atypical symptoms [asthma/chest, pain/cough]: 96.6% vs. 83.9%, *p* = 0.002) [30]. The discrepancies in postoperative symptom control between PPI responders and non-responders may be due to the preoperative failure to discern whether the symptoms are truly caused by reflux or functional disorders [27]. Therefore, it is crucial to differentiate other functional esophageal disorders from proven GERD, including erosive esophagitis (ERD), non-erosive reflux disease (NERD), and reflux hypersensitivity. This can be achieved through endoscopy and esophageal pH monitoring with acid exposure time and DeMeester score.

## 4. Comparison between ARS and PPI

In previous meta-analyses of RCTs comparing ARS and PPI in patients with proven GERD, there were no significant differences or favorable outcomes in patients treated with ARS compared to patients treated with PPI in terms of GERD control, whereas there were no significant differences or inferior outcomes in terms of post-treatment complications [13,35]. Tristão et al. reported that patients undergoing fundoplication showed superior outcome in terms of heartburn remission (risk differences (RD) = −0.19, *p* = 0.0003) and comparable outcome in terms of remission rate of regurgitation (RD = −0.07, *p* = 0.18), pathologic esophageal acid exposure (pH < 4) (mean differences (MD) = −2.40, *p* = 0.64)), and the presence of dysphagia (RD = 0.04, *p* = 0.26) and other complications after treatment compared to patients with PPI. Garg et al. also reported superior outcome in the frequency of postoperative heartburn (short term: risk ratio (RR) = 0.45, 95% confidence interval (CI) 0.30 to 0.69 and long term: RR = 0.59, 95% CI 0.44 to 0.72))) and other reflux symptoms remission (short term: RR = 0.10, 95% CI 0.05 to 0.24) as well as improvements in health-related quality of life (short-term standardized mean difference (SMD) 0.14, 95% CI −0.02 to 0.03)). However, the prevalence of post-treatment dysphagia (short term RR 3.58, 95% CI 1.91 to 6.71) and serious adverse events (short term RR 1.46, 95% CI 1.01 to 2.11) was higher in the surgery group compared to PPI [35].

Though there are only limited studies comparing ARS and PPI in reflux hypersensitivity and other functional esophageal disorders, a previous RCT reported that 71% of patients with reflux hypersensitivity who received ARS experienced symptom improvement at 1 year after treatment initiation, whereas only 62% of patients with proven GERD who received ARS, 28% in the medical treatment group, and 12% in the placebo group achieved symptom improvement [27].

## 5. Cost-Effectiveness of ARS

The economic burden of surgical treatment is less than or comparable to that of medication depending on the treatment period. A nationwide study conducted between 2007 and 2016 compared the characteristics of ARS with PPI treatment in terms of medical expenditure, including PPI, inpatient, and outpatient costs [36]. In the first postoperative year, the costs were ten times higher in the ARS group than in the other groups. However, their costs declined as the follow-up period increased, whereas costs in the PPI group did not. Regarding medical utilization, the ARS group had fewer outpatient and emergency visits. In a cross-sectional analysis of nationwide data between 2012 and 2016 [37], the medical costs within 90 days in the ARS group were 16.9 times higher than those in the PPI group. However, this difference was not significant after 90 days of postoperative follow-up. Park et al. compared the cost- and quality-adjusted life-years (QALYs) of long-term medical and surgical therapies [38] in a cohort of patients with severe GERD who required a continuous double dose of PPI or surgical treatment. Among patients with severe GERD, ARS was more cost-effective than PPI for over 10 years. The model predicted that, compared with the PPI group, the ARS group would have cost savings of $551 and a gain of 1.18 QALYs. The break-even point in the costs of ARS over PPI was estimated at 9 years, which is consistent with the findings of previous studies [17,18,27]. Further studies in a well-selected population with proven GERD and reflux hypersensitivity are required.

## 6. Patient Selection of ARS

**Figure 1:**
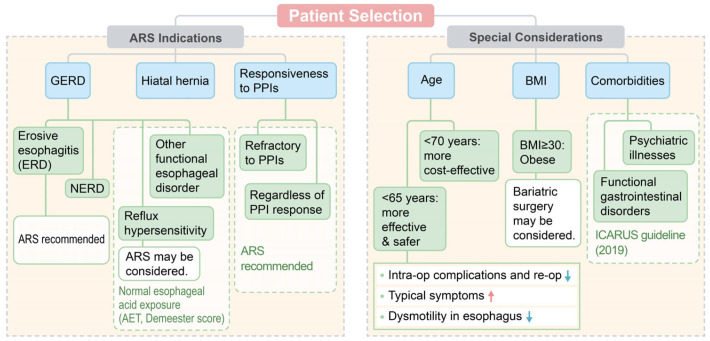
Indications for ARS and areas of special consideration for patient selection.

ARS can be an effective treatment for refractory GERD, with its desirable outcomes and cost-effectiveness. However, it is important to select appropriate candidates for ARS, considering the relatively low surgical effectiveness in PPI non-responders compared to PPI responders, irreversibility of surgery, and possible complications after ARS. Figure 1 shows the current indications for ARS and the areas of special consideration in patient selection.

### 6.1. Association between Symptoms and Reflux Events

Previous guidelines and consensuses recommended ARS as an alternative to PPI maintenance therapy [2,3,16,34,39]. This was applied to patients with abnormal esophageal exposure in a 24 h esophageal study, which included ERD and NERD, after excluding esophageal motility disorders using esophageal manometers. However, recent studies have shown that ARS may be effective in patients with reflux hypersensitivity, defined as a high symptom index (>50%) or symptom-associated probability (>95%), which are indicators of the association between symptoms and reflux events, though they do not meet the diagnostic criteria for GERD [27,28,29,40]. Reflux hypersensitivity can be a possible indication of ARS. However, because of the limited evidence on this issue, further studies are needed.

### 6.2. Age

Several studies have suggested that younger patients may benefit more from ARS than older patients. ARS in patients aged >65 years has been reported to be safe in some studies, whereas others have reported a high rate of intraoperative complications and postoperative mortality [41,42,43,44]. In particular, patients aged >75 years had a higher risk of intraoperative complications (odds ratio (OR) 2.94, *p* = 0.003)) and reoperations (OR 2.36, *p* < 0.05), along with longer operation times (ß 6.29, *p* < 0.001) and lengths of hospital stay (ß 0.56, *p* < 0.001) [45]. Patients < 65 years have more typical symptoms and less esophageal dysmotility than those aged 65 years or older [46]. ARS has greater efficacy in patients with typical symptoms; therefore, expanding the candidates for ARS from younger to older patients seems reasonable.

Regarding cost-effectiveness, in a nationwide study comparing the characteristics of ARS to PPI treatment [36], ARS was cheaper than medication in all age groups except for those aged 70–79 years. In particular, at the 1-year postoperative follow-up, patients aged 20–40 years who underwent ARS incurred one-tenth of the monthly medical costs compared with their counterparts in the PPI group. Moreover, young people may be required to continue PPIs for their lifetime, which could pose a high financial burden and expose them to the long-term adverse effects of PPIs.

### 6.3. Body Mass Index

Several studies have shown that an elevated body mass index (BMI) is associated with GERD [47,48]. However, studies have shown conflicting clinical outcomes of ARS in patients with obesity. Fraser et al. [49] reported that a higher BMI was not correlated with poorer outcomes when patients were divided based on BMIs (normal weight: BMI < 25 kg/m^2^; overweight: BMI 25–29.9 kg/m^2^; and obesity: BMI > 30 kg/m^2^). Luketina et al. [50] reported that obesity was not correlated with poor postoperative outcomes when patients were divided into two groups based on BMI (normal weight: BMI 20–25 kg/m^2^; and obesity: BMI ≥ 30 kg/m^2^). A meta-analysis by Tandon et al. [51] reported that the rates of procedural conversion and reflux recurrence requiring reoperation (RR 1.99, *p* = 0.11) or wrap migration (RR 1.23, *p* = 0.73) were similar between the obesity and non-obesity groups. However, different meta-analyses reported that patients with obesity (BMI ≥ 30 kg/m^2^) have higher reflux recurrence rates than patients without obesity [52,53]. Therefore, gastric bypass, which is one of the most frequently performed bariatric surgeries, could be considered an alternative to ARS in patients with severe obesity [54,55].

In contrast to gastric bypass, the effect of sleeve gastrectomy, one of the other most widely performed bariatric surgeries, on GERD is controversial [56,57]. In previous studies, sleeve gastrectomy was considered to exacerbate GERD due to weakening of the anti-reflux barrier, including decreased LES pressure and blunting of the angle of His, as well as the decrease in gastric compliance and volume leading to an increase in gastric pressure [58,59,60,61]. In contrast, other studies have shown that sleeve gastrectomy improves GERD due to increased gastric emptying and decreased acid secretion [56,62,63]. Therefore, a new surgical approach combining sleeve gastrectomy and fundoplication is being studied as a novel surgical procedure to obtain the benefits of both surgeries to address both severe obesity and GERD simultaneously [64].

Since BMI differs between Western and Asian populations, parameters for Asian populations must be established to select appropriate candidates. In terms of bariatric surgery and metabolic surgery, a guideline from American Society of Metabolic and Bariatric Surgery (ASMBS) and International Federation for the Surgery of Obesity and Metabolic Disorders (IFSO) suggested that the BMI thresholds of metabolic and bariatric surgery in the Asian population should be adjusted to 25–27.5 kg/m^2^ considering the higher prevalence of diabetes and cardiovascular diseases in Asian populations with lower BMI than in non-Asian populations [65]. However, no previous studies have investigated the efficacy of bariatric surgery compared with ARS in Asians with a BMI of 25–30 kg/m^2^, which is considered non-obese (considered as overweight) in the Western population but obese in the Asian population. Future studies are warranted to determine whether BMI affects surgical outcomes in patients with a BMI of 30 > BMI ≥ 25 kg/m^2^ and whether bariatric surgery is also effective within this BMI range.

### 6.4. Comorbidities

The International Consensus regarding preoperative examinations and the assessment of clinical characteristics for selecting adult patients for Anti-Reflux Surgery (ICARUS) guidelines recommend that patients with GERD and comorbidities are good indications for ARS. These can include functional disorders such as dyspepsia and irritable bowel syndrome, and psychiatric illnesses including major depressive disorder or anxiety disorder, if these symptoms can be attributed to reflux [39]. However, further studies are required to determine the extent to which these comorbidities should be considered before performing ARS.

## 7. Complications and Failure of ARS

A meta-analysis of RCTs in patients with ARS experienced dysphagia (27%), inability to belch (31%), gas bloating (18%), and flatulence (25%) at 1-year follow-up after ARS [66]. Similarly, a nationwide cohort study in Korea performed from 2011 to 2018 [67] investigated the incidence of four postoperative complications: moderate-to-severe dysphagia (23.5%), inability to belch (29.4%), gas bloating (23.2%), and flatulence (22.0%) at 1-year follow-up.

The failure and reoperation rates of ARS were reportedly up to 30% and 5–8%, respectively [68,69,70,71]. Common causes for reoperation include recurrent reflux, dysphagia, and paraesophageal hernia, which are usually secondary to wrap herniation or slippage [72,73,74]. The most common anatomical abnormalities are slipped fundoplication, wrap malpositioning, intrathoracic wrap migration, and complete or partial wrap disruption [71,75,76]. Although the success rates for revision ARS were lower than those for primary ARS, more than 80% of the patients were satisfied with the reoperation outcomes [77]. The wrap appeared loose during revision surgeries and partial thinning of the anterior wrap was observed in most patients with recurrent reflux symptoms. However, the factors affecting the wrap durability remain unclear and require further studies [78]. Therefore, there is an ongoing need to enhance the efficacy and safety of ARS.

Optimal approaches to reverse operation failure should be assessed. Though there is limited data available to guide the management of patients who have undergone two or more prior failed ARS procedures, several studies have investigated revision surgery for ARS, including redo fundoplication, hernia repair with mesh reinforcement, Collis gastroplasty, and Roux-en-Y gastric bypass [79]. According to a previous meta-analysis, laparoscopic redo fundoplication had a conversion rate of 6.02% and a major morbidity rate of 4.98%. Regarding effectiveness, laparoscopic redo fundoplication resulted in symptom improvement in 78.5% of patients and enhanced quality of life in 80.65% of patients. However, GERD recurrence after the procedure occurred in 10.71% of the cases [80]. Meanwhile, a different meta-analysis focused on Roux-en-Y gastric bypass as a revisional bariatric surgery following failed ARS in severe obesity and found that the symptom improvement rate after revision Roux-en-Y gastric bypass was 92.62%, with a perioperative complication rate of 16.7% [81]. Based on current evidence, redo fundoplication can be an effective procedure for revision surgery after ARS failure and Roux-en-Y gastric bypass can be considered an alternative to redo fundoplication in patients with severe obesity.

Structured training protocols and programs should be implemented to shorten the learning curves for ARS in surgeons. Studies have shown that surgeons must perform approximately 20 procedures to overcome the learning curve for ARS [82,83]. However, dangerous complications occurred until the sixtieth procedure, according to a learning curve study conducted in Germany [84]. Proctorships and careful mentoring by experienced surgeons can accelerate the technical maturation of novice surgeons. One study demonstrated that trainees with little ARS experience achieved tolerable outcomes when adequately supervised [85].

## 8. Nissen Fundoplication and Partial Fundoplication

Nissen fundoplication is the most commonly performed ARS procedure. However, partial fundoplication is also performed to prevent post-operative dysphagia, bloating, and other complications. Two types of partial fundoplication are usually performed: Dor fundoplication or anterior fundoplication and Toupet fundoplication or posterior fundoplication.

Previous meta-analyses of RCTs comparing Nissen fundoplication and Dor fundoplication found that the frequency of postoperative dysphagia (RR 0.56, *p* = 0.002), flatulence (RR 0.57, *p* = 0.02), inability to belch (RR 0.63, *p* = 0.05), and gas bloating (RR 0.59, *p* < 0.001) was significantly higher in the Nissen fundoplication group than in the Dor fundoplication group. However, there were no significant differences in postoperative heartburn (RR 1.39, *p* = 0.58) and PPI use [66]. Postoperative DeMeester scores (weighted mean difference (WMD) 0.85, *p* = 0.06), LES pressure (SMD −0.74 mmHg, *p* = 0.23), and reoperation rates (RR 1.50, *p* = 0.24) also showed no significant differences between the two procedures [86].

Similarly, in a previous meta-analysis of RCTs comparing Nissen and Toupet fundoplication, postoperative dysphagia (RR 2.61, *p* < 0.01) and gas-related symptoms (RR 1.31, *p* = 0.02) including the inability to belch, gas bloating, and flatulence were significantly more frequent in patients undergoing Nissen fundoplication than in those who underwent Toupet fundoplication. Though patient satisfaction (RR = 1.05, *p* = 0.22) and reoperation rates (4.74% vs. 6.54 %, *p* = 0.77) did not significantly differ between the two groups, postoperative LES pressure was higher (SMD 0.66, *p* < 0.05) and the postoperative DeMeester score (SMD −0.72, *p* = 0.06) was not statistically significant but lower in the Nissen fundoplication group than in the Toupet fundoplication [87].

In a meta-analysis of previous RCTs comparing Dor and Toupet fundoplication, the postoperative dysphagia score (WMD −2.87, *p* < 0.001) was lower in the Dor fundoplication group. However, the esophageal acid exposure time (WMD 2.04%, *p* < 0.001), postoperative heartburn rate (RR 2.71, *p* < 0.001), and reoperation rate (RR 2.12, *p* = 0.03) were higher in the Dor fundoplication group than in the Toupet fundoplication group. There were no significant differences between Dor fundoplication group and Toupet fundoplication group in terms of regurgitation (RR 1.09, *p* = 0.90), inability to belch (RR 0.52, *p* = 0.14), and gas bloating (RR 0.76, *p* = 0.07) [88].

Therefore, Nissen fundoplication may show better efficacy than partial fundoplication in controlling reflux symptoms. However, considering that postoperative symptoms such as dysphagia and gas bloating may occur frequently, recent guidelines suggest that the choice of surgical procedure should be based on whether the patient prioritizes symptom improvement or minimizing postoperative symptoms such as dysphagia [16].

## 9. Public and Health Workers’ Awareness of ARS

Several barriers to creating awareness about ARS among patients and healthcare workers have hindered its optimal utilization. Most patients with GERD receive PPI therapy because they are unaware of ARS as a treatment option and believe that GERD can only be treated with medications. Another prevailing misconception is the consideration of surgery as a last resort among available treatment modalities. Considering the accumulating clinical evidence of ARS in patients with various conditions, it is reasonable to doubt whether refractoriness to medication is the only appropriate indication for surgery. A better understanding of the safety and efficacy of the ARS could help patients and healthcare workers identify appropriate surgical candidates, which would contribute to optimizing treatment and improving the quality of life of patients with GERD. Furthermore, some patients are reluctant to undergo ARS because of fear of adverse effects, such as dysphagia, gas bloating, and difficulty in vomiting [89]. However, dysphagia is usually self-limiting and is not associated with worse long-term GERD symptom control [90]. In addition to laparoscopic surgery, minimally invasive surgery is expected to dispel patients’ concerns regarding invasiveness. Considering these gaps in the understanding of the real aspects of ARS, we must evaluate patients’ and physicians’ understanding to lessen the gaps.

Therefore, healthcare workers with patient-tailored attitudes are warranted. In addition to age and BMI, it is necessary to emphasize patient preferences, desires, and values when choosing treatment options from medication to surgery. Based on this understanding, patients can choose more suitable treatment options and communicate better with healthcare providers [91].

## 10. Conclusions

The incidence of GERD has rapidly increased, with most patients being treated with medications, resulting in an alarming rate of PPI prescriptions. The present article comprehensively reviewed the efficacy and cost-effectiveness of ARS compared to medications, and other factors that guided the selection of candidates for ARS. ARS is widely recognized as an effective and safe treatment option for GERD, but it is rarely performed. A careful evaluation with endoscopy or pH monitoring is necessary to select patients with ‘proven GERD’ and, with limited evidence, reflux hypersensitivity. Regarding cost-effectiveness, recent studies have demonstrated that the medical costs for ARS were equal to those for PPI maintenance treatment after 9 years of follow-up. Considering the growing disease burden on patients, appropriate surgical treatment should be implemented according to emerging clinical guidelines. Further research is needed to determine the patient selection criteria regarding age, BMI, and comorbidities. Efforts to reduce and manage postoperative complications and promote awareness of ARS safety and durability are warranted, along with adequate training programs for younger surgeons.

## Data Availability

Not applicable.
